# Polyacrylamide-Phytic Acid-Polydopamine Conducting Porous Hydrogel for Efficient Removal of Water-Soluble Dyes

**DOI:** 10.1038/s41598-017-08220-6

**Published:** 2017-08-11

**Authors:** Zhen Zhao, Leijiao Li, Girma Selale Geleta, Lina Ma, Zhenxin Wang

**Affiliations:** 1 State Key Laboratory of Electroanalytical Chemistry, Changchun Institute of Applied Chemistry, Chinese Academy of Sciences, Changchun, 130022 P. R. China; 2 University of Chinese Academy of Sciences, Beijing, 100049 P. R. China; 3 State Key Laboratory of Rare Earth Resource Utilization, Changchun Institute of Applied Chemistry, Chinese Academy of Sciences, Changchun, 130022 P. R. China

## Abstract

Removal of toxic dyes from wastewater has become a hot topic in both academic and industrial fields since there is growing concern about the threat of sewage to human health. Herein, we demonstrate that the three-dimensional porous polyacrylamide-phytic acid-polydopamine (termed as PAAM/PA/PDA) hydrogel can be served as reusable adsorbent with high efficiency for either anionic or cationic dyes. Using methyl blue (MB), methylene blue (YMB), methyl violet (MV) and neutral red (NR) as model dyes, we investigate the effect of pH, temperature, dye concentration, and PAAM/PA/PDA hydrogel mass on the adsorption. The experimental maximum adsorption capacities are more than 350.67 mg g^−1^ for four selected dyes. Adsorption kinetic and thermodynamic analysis suggests that the dyes are adsorbed on the PAAM/PA/PDA hydrogel through the strong π-π stacking and anion-cation interaction, and the adsorption process satisfies a pseudo-second-order model. Furthermore, the free-standing PAAM/PA/PDA hydrogel can be easily removed from water after adsorption process, and regenerated by adjusting solution pH values.

## Introduction

The industrial activities including the manufacture of leather, cosmetic, textile, paper and paint, as well as food processing produce massive amount of waste water containing toxic and carcinogenic dyes that pollute water intended for human consumption^1–6^. The phenomenon cause a great challenge to public health and has been linked to serious diseases including cancer and cardiovascular disease. There are more than a hundred thousand commercially available dyes which are discharged into water bodies^7^. However, most of industrial dyes are difficult to remove/degrade from water-bodies because they normally have high physicochemical stability.

Various techniques have been employed for treatment of dye-containing wastewater, including catalytic degradation^1^, ion exchange^8^, membrane separation^9^, coagulation-flocculation^10^, precipitation^11^, photo degradation^12^, biological degradation^13^ and adsorption^14–23^, *etc*. Among these methods, adsorption is considered to be one of the most effective methods for its simplicity, high efficiency, flexibility, and insensitivity to toxic substance^14, 15^. Many adsorbents such as activated carbon, mesoporous silica, magnetic nanoparticles, boron nitride nanosheets, graphene oxide/graphene hydrogels and agricultural solid wastes have been used to remove the dyes from wastewater^14–21, 24–29^. Although these adsorbents exhibit high adsorption capacity and efficiency, most of them are high cost and limited only to adsorb cationic dyes or anionic dyes. It is still a challenge to prepare high efficiency, reusable and broad-spectrum adsorbents. Recently, polymeric hydrogels have been considered as high performance materials for environmental applications such as desalination^30^, heavy metal ions removal^31^ and radionuclides^32^ due to their advantageous characteristics including adsorption-regeneration ability, economic feasibility, and environmental compatibility. However, there are few of studies on the removal of dyes using polymeric hydrogels.

In our previous experiment, the PAAM/PA/PDA hydrogel has been synthesized and employed to prepare reusable electrochemical sensor and absorbent for monitoring and removing Cu^2+^ in real samples. In this study, the PAAM/PA/PDA hydrogel is used as environmental pollutant scavenger to adsorb either anionic or cationic dyes with aromatic structure by tuning the surface charge of the hydrogel via pH value. The PAAM/PA/PDA hydrogel exhibits relative high adsorption capability within 300 minutes, and could be reused several times without significant decrease (less than 4.32%) in adsorption capability. The results demonstrate that the PAAM/PA/PDA hydrogel has great potential to be used as a kind of fast, efficient broad-spectrum adsorbent for remediation of dye polluted water bodies.

## Results

### Swelling measurement of PAAM/PA/PDA hydrogel

As shown in Fig. S1, the swelling ratio of PAAM/PA/PDA hydrogel increases with the increasing time until reaching equilibrium. The value of equilibrium swelling ratio is about 632.15%. It is well known that the swelling of hydrogel is induced by the electrostatic repulsion of the ionic charges of its network^33^. The high equilibrium swelling ratio is due to the abundant functional groups (-COOH, -OH) of PAAM/PA/PDA hydrogel.

### Optimization and evaluation of dye adsorption capacity

**Figure 1:**
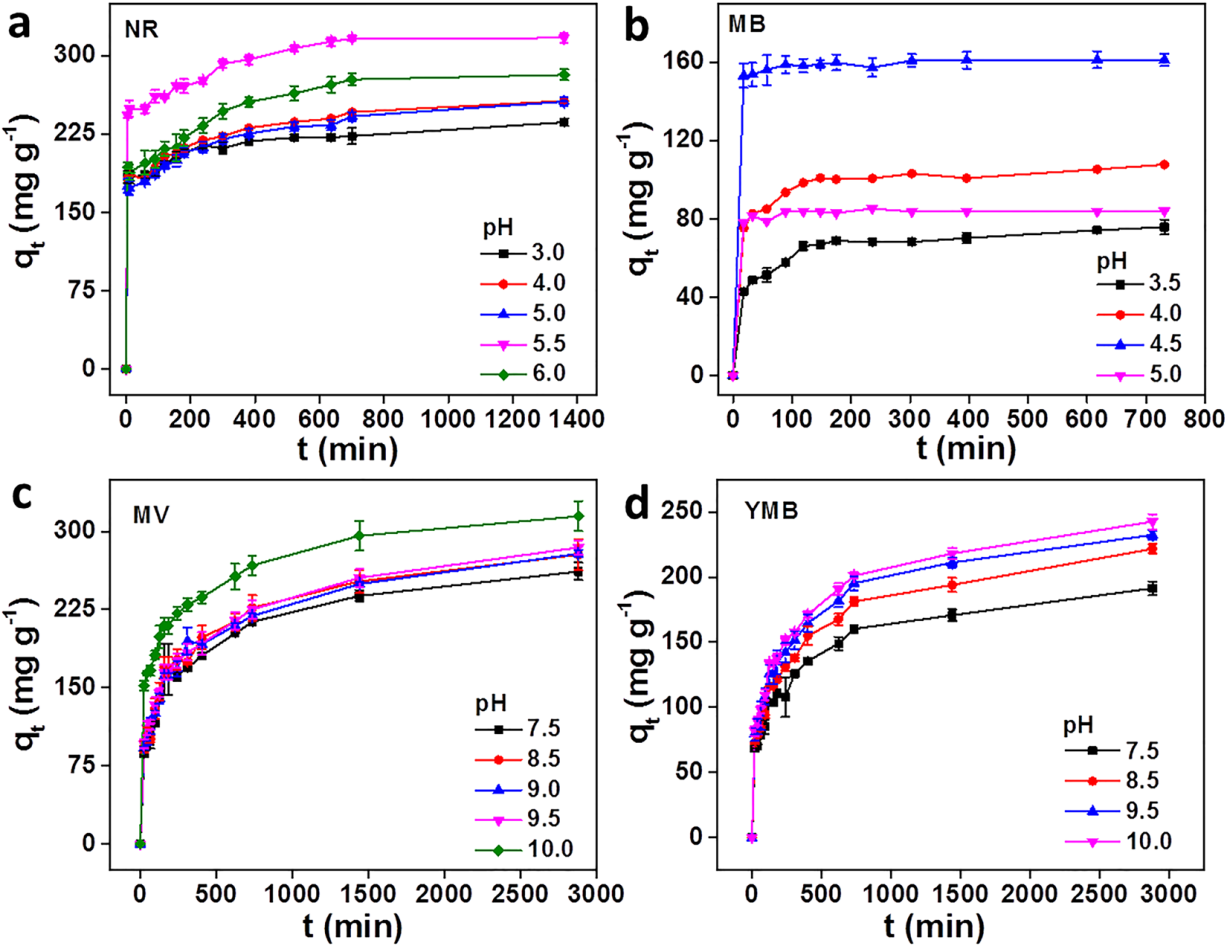
The effect of pH value on the adsorption of PAAM/PA/PDA hydrogel to (**a**) NR, (**b**) MB, (**c**) MV and (**d**) YMB, respectively.

The effects of temperature, pH value, adsorbent dosage and initial concentration of dyes on the adsorption capacity have been investigated. The surface charge of the PAAM/PA/PDA hydrogel is strongly dependent on the solution pH value and the zero point charge (pzc) of PAAM/PA/PDA hydrogel is calculated as pH 5.3 (as shown in Fig. S2). As shown in Fig. 1, the negatively charged PAAM/PA/PDA hydrogel exhibits high equilibrium absorption capacities for cationic dyes (NR, MV, and YMB) while positively charged PAAM/PA/PDA hydrogel has high equilibrium absorption capacity for anionic dye (MB). The maximum adsorption capacities were 317.05 mg g^−1^ for NR at pH 5.5, 161.18 mg g^−1^ for MB at pH 4.5, 314.55 mg g^−1^ for MV at pH 10.0 and 242.46 mg g^−1^ for YMB at pH 10.0, respectively. The experimental result indicates that electrostatic interaction plays important role in the dye adsorption. The phenomenon is consistent with the literature report^34^.

As shown in Fig. S3, the equilibrium absorption capacities are decreased from 1008.65 to 298.64 mg g^−1^ for NR, 393.25 to 168.82 mg g^−1^ for MB, 810.67 to 251.30 mg g^−1^ for MV and 553.37 to 224.93 mg g^−1^ for YMB with increasing PAAM/PA/PDA hydrogel dosage from 2.5 to 10.0 mg, respectively, while the concentrations of the dyes are kept as constants. In contrast, the adsorption efficiencies are increased from 65.5% to 92.5% for NR, 51.8% to 93.2% for MB, 51.3% to 89.3% for MV and 54.9% to 91.5% for YMB with increasing hydrogel dosage from 2.5 to 10.0 mg, respectively. This phenomenon can be explained by adsorption equilibrium between dye and PAAM/PA/PDA hydrogel^35–37^. Large dosage of PAAM/PA/PDA hydrogel can provide high amount of adsorption sites, resulting in decreased significantly the concentration of free dye in the solution. The utilization rate of adsorption sites is decreased by the low concentration of free dye, which leads to low equilibrium absorption capacity.

Figure S4 demonstrates the effect of initial concentrations of dyes on the equilibrium absorption capacity while dosage of PAAM/PA/PDA hydrogel is kept as constant. As can be seen, with the increasing concentrations of dyes from 5 to 50 mg mL^−1^, the maximum adsorption capacities of PAAM/PA/PDA hydrogel are increased to 566.61, 381.35, 529.96 and 341.29 mg g^−1^ for NR, MB, MV and YMB while the adsorption efficiencies are decreased to 91.4%, 92.1%, 87.9% and 90.2% for NR, MB, MV and YMB, respectively. This phenomenon can be explained as the high driving force for mass transfer and the saturation of the active adsorption sites, respectively^36, 37^.

As shown in Fig. S5, with the increasing adsorption temperature from 285 K to 318 K, the adsorption capacities of PAAM/PA/PDA hydrogel are increased from 550.59 to 584.58 mg g^−1^ for NR, 332.06 to 405.99 mg g^−1^ for MB, 485.92 to 625.97 mg g^−1^ for MV and from 280.97 to 350.67 mg g^−1^ for YMB, while the adsorption efficiencies are increased from 87.9% to 93.3% for NR, 79.5% to 96.9% for MB, 70.5% to 95.9% for MV and 77.6% to 94.7% for YMB, respectively. This phenomenon indicates that the thermal energy is another critical factor of the adsorption process. High thermal energy can enhance the diffusion abilities of dye molecules, resulting in the high saturated equilibrium levels. The result confirms the endothermic nature of the dye adsorption on PAAM/PA/PDA hydrogel^20, 38^. Therefore, under optimized conditions (318 K adsorption temperature, 5 mg PAAM/PA/PDA hydrogel in 8 mL solution, 50 mg L^−1^ initial dye concentration, and suitable pH values (5.5 for NR, 4.5 for MB, 10.0 for MV and YMB)), the maximum adsorption capacities (584.58 mg g^−1^ for NR, 405.99 mg g^−1^ for MB, 625.97 mg g^−1^ for MV, and 350.67 mg g^−1^ for YMB) can be obtained respectively. The maximum adsorption capacities are comparable to or higher than those of literature reported (as shown in Table 1)^14–23, 39–49^.

**Table 1:** Maximum adsorption capacities of various adsorbents toward NR, MB, MV and YMB.

dyes	adsorbents	Q_max_ (mg g^−1^)	Ref.
NR	metal-organic framework	132.00	40
	three-dimensional graphene	175.00	41
	smart adsorbents	20.00	42
	Mn-impregnated activated carbons	285.70	43
	PAAM/PA/PDA hydrogel	584.58	this work
MV	GO sponge	467.00	18
	h-XG/SiO_2_ nanocomposite	378.80	42
	metal organic gels	56.00	44
	chitosan aniline/peanut hull	105.20	45
	PAAM/PA/PDA hydrogel	625.97	this work
MB	Ni-MgO	367.00	33
	HPP-NH_3_^+^	449.00	50
	magnetic nanoparticles	136.00	47
	Ni-MCM-41	189.10	48
	PAAM/PA/PDA hydrogel	405.99	this work
YMB	boron nitride nanosheets	313.00	19
	GO-based hydrogels	323.90	21
	agricultural solid wastes	64.40	23
	Hem/GH	99.20	49
	PAAM/PA/PDA hydrogel	350.67	This work

## Discussion

**Figure 2:**
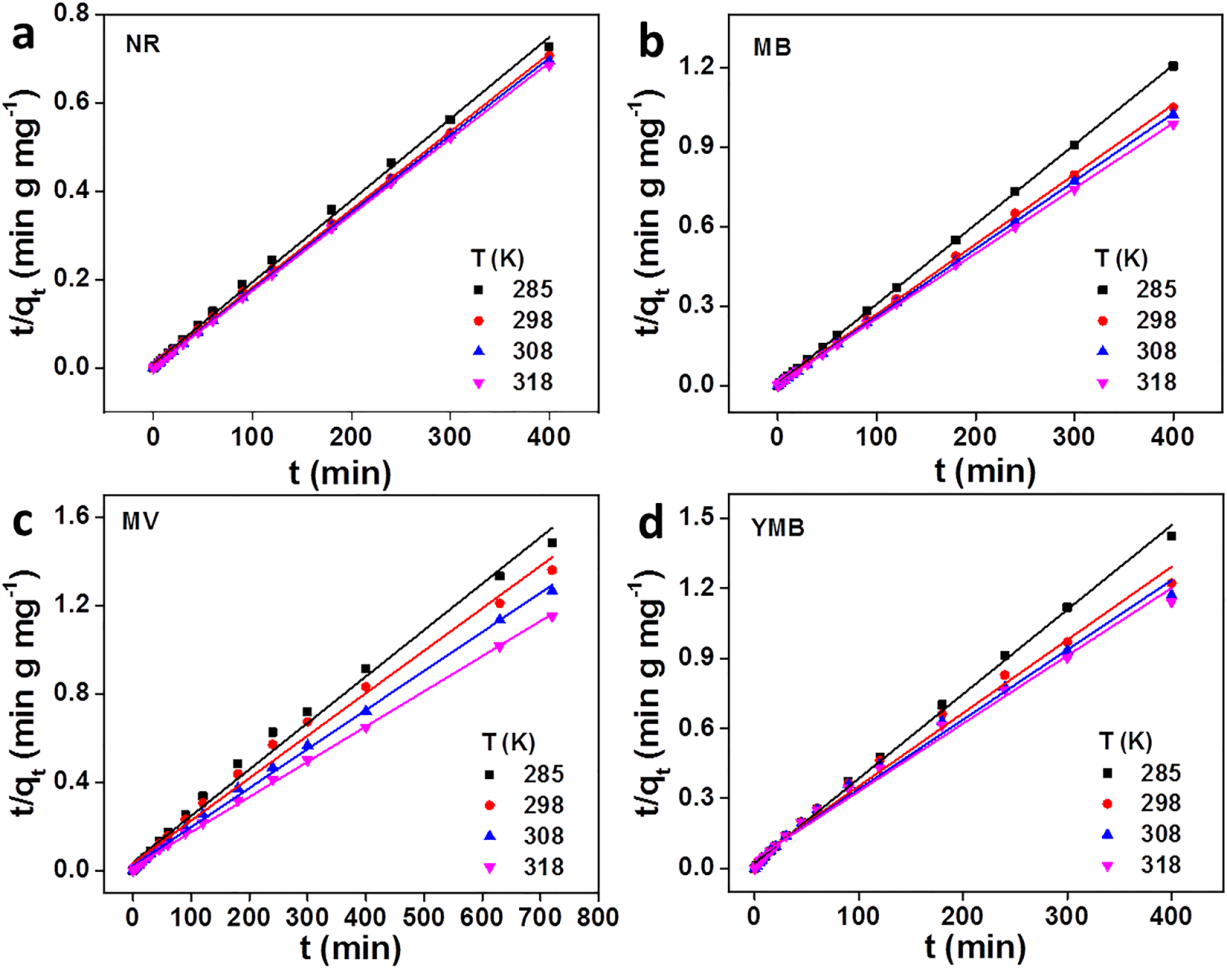
Modeling of the adsorption kinetics of (**a**) NR, (**b**) MB, (**c**) MV and (**d**) YMB using pseudo-second-order models onto PAAM/PA/PDA hydrogel.

It is well known that the rate of dye adsorption depends on the contact time of the solid and liquid as well as on the diffusion processes^50^. During adsorption process, dye molecules migrate to the surface of the PAAM/PA/PDA hydrogel firstly, and then diffuse in the boundary layer and eventually diffuse from the hydrogel surface into the internal sites via pore diffusion. The pseudo-first-order, pseudo-second-order and intra-particle diffusion models were investigated for analyzing the adsorption kinetics (see Supporting Information 2.2 for details). The fits of the experimental results (as shown in Tables S2,S3) show that the pseudo-second-order model possesses a higher R^2^ value, compared with the pseudo-first-order model (as shown in Fig. S6). This phenomenon indicates that the adsorption kinetics can be described as a pseudo-second-order model (as shown in Fig. 2) which depends on the amount of dyes adsorbed on the surface of hydrogel and the amount adsorbed at equilibrium^36^.

Figure S7 presents the intra-particle diffusion kinetics model of Weber-Morris^51^, and the fits of the experimental results are given in Table S4. There are two linear slopes in each curve, suggesting that at least two steps are involved during adsorption process. The first linear slope represents boundary layer diffusion attributed to the transfer of dye to the surface of the PAAM/PA/PDA hydrogel. The second linear slope indicates a steady adsorption step, corresponding to intra-particle diffusion of dye molecules throughout the porous surface of the hydrogel^51, 52^. In addition, there is the thickness of the boundary layer since the curves possess intercepts^53^. The presence of multilinearity and the boundary layer thickness suggests that surface adsorption may also play significant role in the dye uptake process in combination with intra-particle diffusion model.

**Figure 3:**
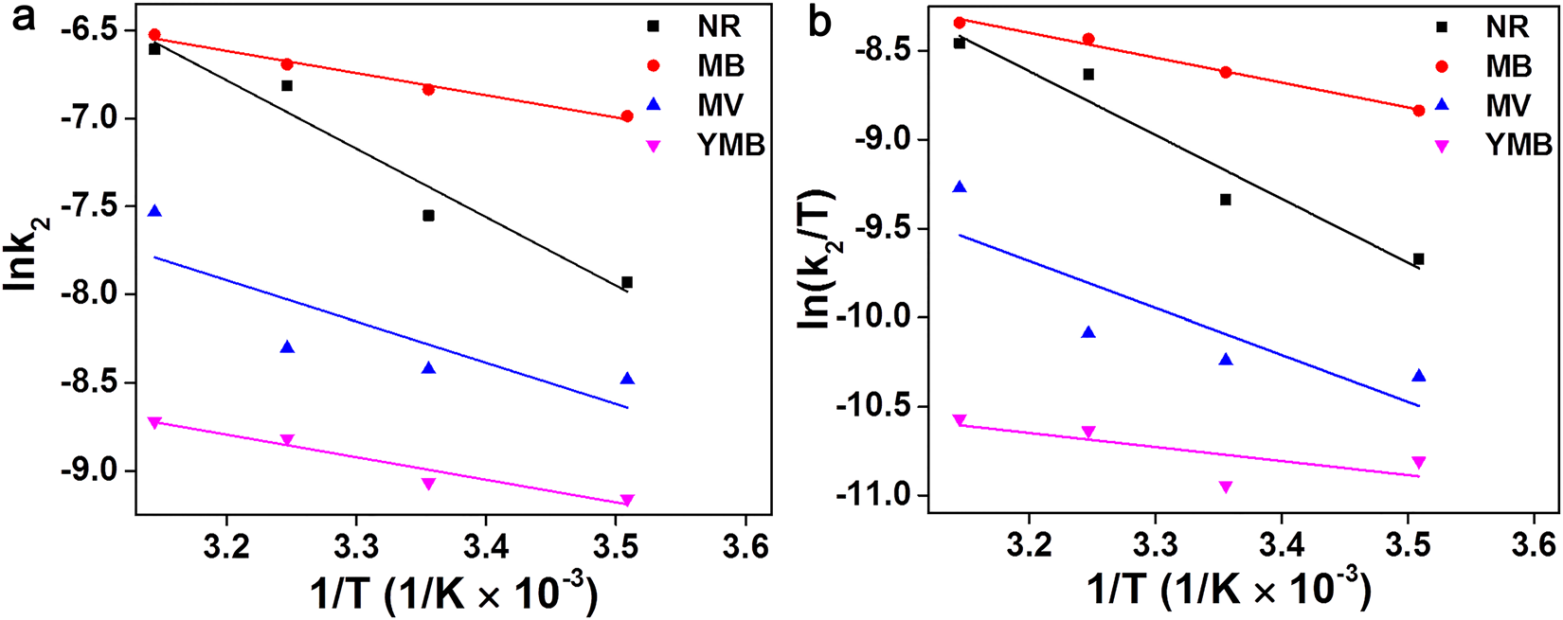
Plots of (**a**) Arrhenius and (**b**) Eyring for NR, MB, MV and YMB adsorption on the PAAM/PA/PDA hydrogel, respectively.

The thermodynamic parameters of dyes on PAAM/PA/PDA hydrogel were analyzed by Arrhenius equation and Eyring equation (see Supporting Information 2.3 for details). As shown in Fig. 3a, the activation energies of NR, MB, MV and YMB are 64.65, 47.18, 75.45 and 67.98 kJ mol^−1^, respectively. The as-obtained activation energies are higher than activation energy of physical adsorption process (*c.a*., 5–40 kJ mol^−1^), suggesting that the dye are interacted with PAAM/PA/PDA hydrogel through chemical adsorption (*e.g*., π-π stacking)^20^. The values of activation Enthalpies are 59.66 (NR), 46.53 (MB), 73.69 (MV) and 66.65 (YMB) kJ mol^−1^, respectively (as shown in Fig. 3b). These results also suggest the adsorptions of dyes with PAAM/PA/PDA hydrogel are endothermic process^54^.

After adsorbed by PAAM/PA/PDA hydrogel, the maximum absorbance wavelengths of NR, MB, MV and YMB are bathochromic shifted about 14, 8, 13 and 15 nm respectively (as shown in Fig. S8), indicating the strong π-π stacking interaction between hydrogel and dyes^55^. As shown in Fig. S9, after adsorbed with dyes, the C = C stretching peak of PAAM/PA/PDA hydrogel is changed from 1674 to 1641 cm^−1^ for NR, 1674 to 1647 cm^−1^ for MB, 1674 to 1642 cm^−1^ for MV, and 1674 to 1646 cm^−1^ for YMB, respectively, also indicating that π-π stacking interaction is involved in adsorption process.

**Figure 4:**
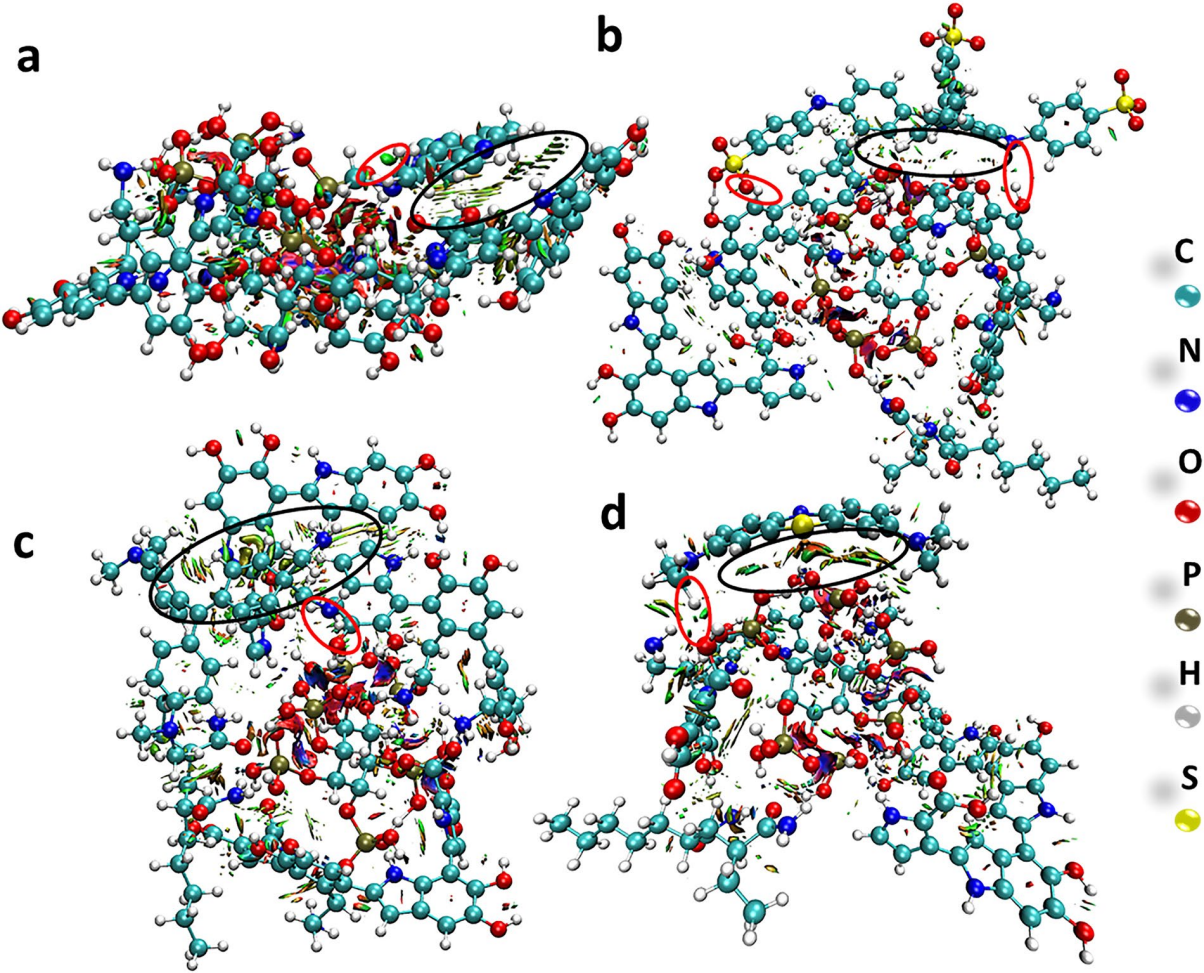
Gradient isosurfaces (*s* = 0.5 au) for (**a**) NR-PAAM/PA/PDA hydrogel, (**b**) MB-PAAM/PA/PDA hydrogel, (**c**) MV-PAAM/PA/PDA hydrogel and (**d**) YMB-PAAM/PA/PDA hydrogel through VMD (Visual Molecular Dynamics)^60^, respectively. The surfaces are colored on a blue-green-red scale according to values of sign(λ_2_)ρ, ranging from −0.04 to 0.02 au. Blue indicates strong attractive interactions, and red indicates strong nonbound overlap. Large and negative values of sign(*λ*_2_)*ρ* indicate attractive interactions (such as dipole-dipole or hydrogen bonding, blue color), while large and positive values of sign(*λ*_2_)*ρ* represent nonbonding (red color). The values of sign(*λ*_2_)*ρ* near zero indicate van der Waals interactions, such as π-π stacking effect.

Semi-empirical PM6-D3H4 method was employed to further figure out the adsorption mechanism between the PAAM/PA/PDA hydrogel and dye molecules by using Mopac2012 program since weak interactions in molecular system includes hydrogen bonding, dipole-dipole interactions, π-π interactions, steric repulsion and London dispersion^56, 57^. The gradient isosurfaces were colored according to the corresponding values of sign(*λ*_2_)*ρ* ^58^, which is found to be a good indicator of interaction strength. The gradient isosurfaces provide a rich visualization of noncovalent interactions as broad regions of real space, rather than simple pairwise contacts between atoms. The reduced density gradient (*s* = 0.5 au) of the dyes-PAAM/PA/PDA hydrogel generated by program Multiwfn^59^. The surfaces are colored on a blue-green-red scale according to values of sign(*λ*_2_)*ρ*, ranging from −0.04 to 0.02 au. The low-gradient isosurfaces for dye molecules adsorbed to PAAM/PA/PDA hydrogel shows the complex webs of noncovalent interactions between the hydrogel and active sites (as shown in Fig. 4). The anion-cation interactions exist in all the dyes-PAAM/PA/PDA hydrogel labeled by red circle, but only part of them can be seen from the Fig. 4. Strong π-π stacking interactions between the dye molecules (NR and MV) and PAAM/PA/PDA hydrogel are clearly manifested by isosurfaces filling the interlayer spaces (as shown in Fig. 4a,c, labeled by black circle), which are stronger than the π-π stacking interactions between MB and hydrogel (as shown in Fig. 4b). As shown in Fig. 4d, steric clashes have been offset by stronger π-π stacking effect and anion-cation interactions forming the YMB-PAAM/PA/PDA hydrogel structure.

**Figure 5:**
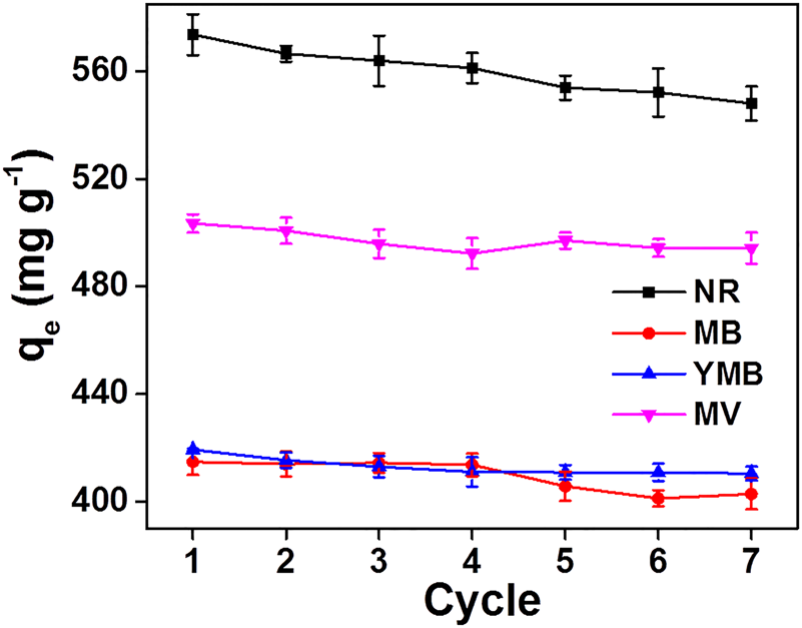
The regeneration of PAAM/PA/PDA hydrogel.

**Figure 6:**
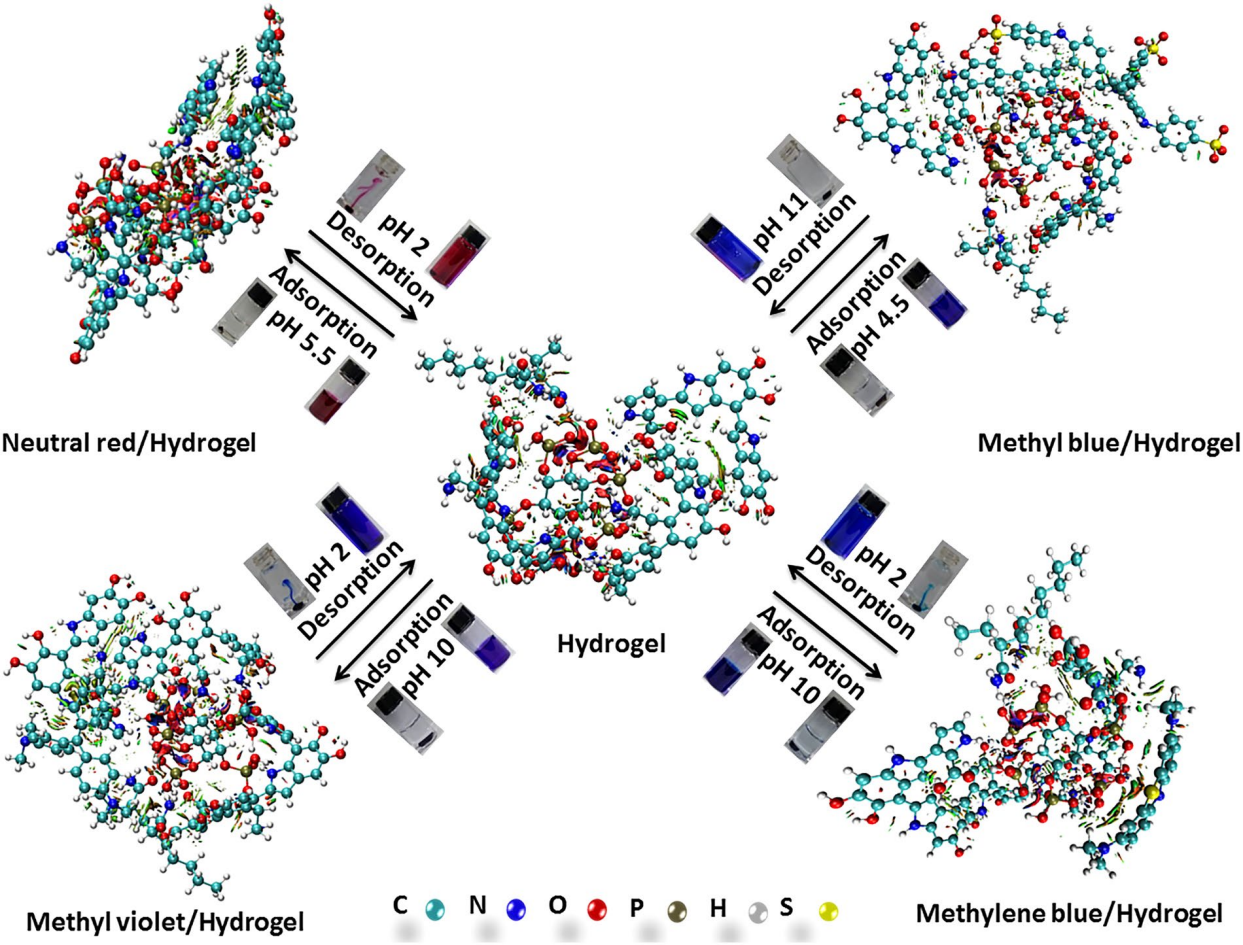
The illustration of dyes adsorption-desorption on PAAM/PA/PDA hydrogel.

For testing it regeneration ability, the adsorption and desorption of dye with the PAAM/PA/PDA hydrogel was performed (as shown in Fig. 5 and Fig. S10) and summarized in Fig. 6. The dye desorption can be easily achieved by adjusting solution pH values (i.e., pH 2.0 for cationic dye (NR, MV and YMB) and pH 11.0 for anionic dye (MB)). After 7 times of adsorption-desorption, there are negligible changes of the equilibrium absorption capacities (as shown in Fig. 5). This result indicates that the dye removal and recovery were stable.

**Figure 7:**
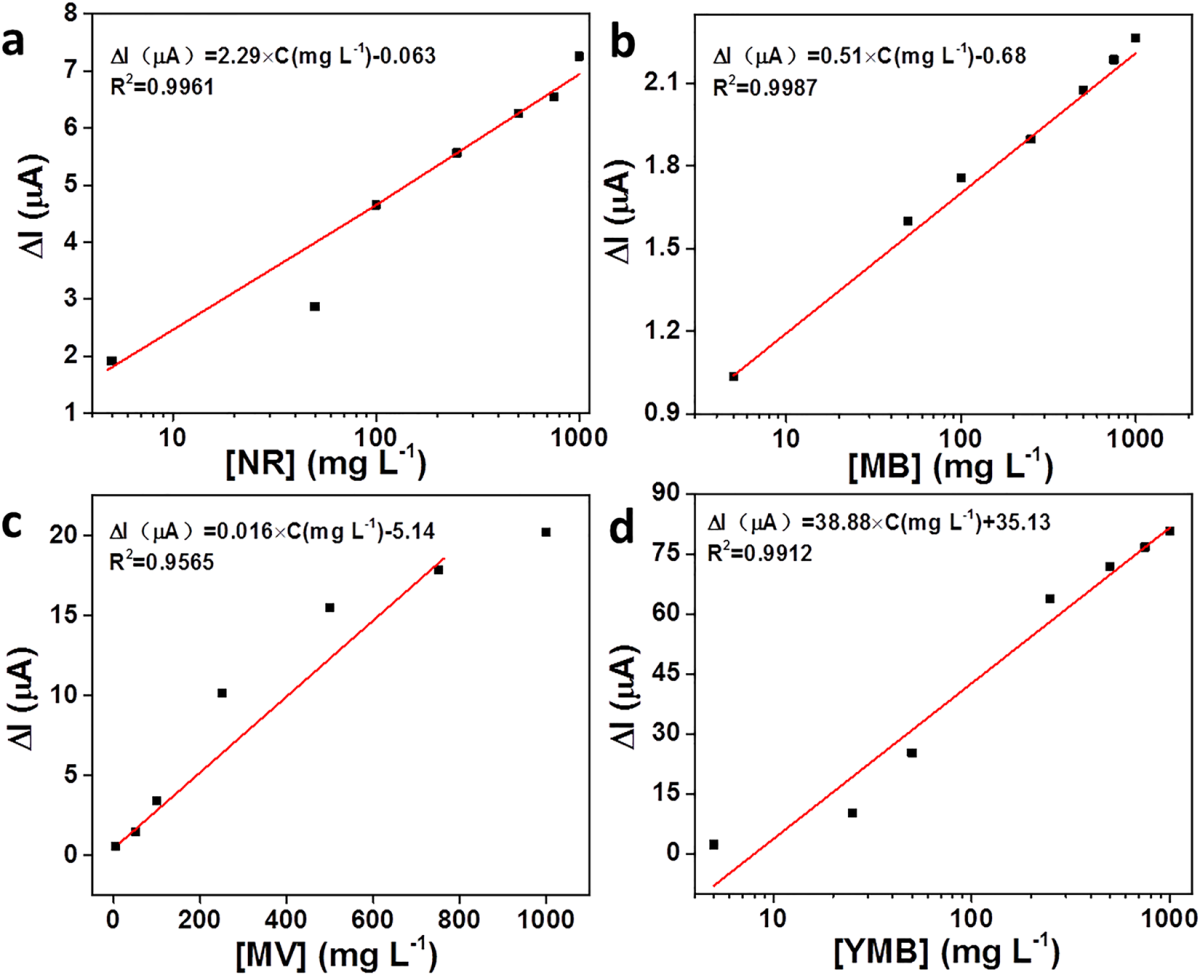
Corresponding plot of the change of maximum peak current (ΔI) as a function of the concentration of (**a**) NR, (**b**) MB, (**c**) MV and (**d**) YMB, respectively. Error bars mean standard deviation (n = 3).

The adsorption/desorption of dyes on the PAAM/PA/PDA hydrogel can be further proved by DPV curves using the PAAM/PA/PDA hydrogel-based electrochemical sensor since the dyes have electrochemical activity. As shown in Fig. 7 and Fig. S11, the peak currents are increased with increasing concentration of dyes. The different dye molecules give different positions of current peaks (−0.60 V for NR, −0.45 V for MB, −0.65 V for MV, and −0.25 V for YMB), respectively, which are agreement with the literature reported values^61–64^. After treated NR, MV, YMB at pH 2.0 and MB at pH 11.0, no current peaks appear on the DPV curves, indicating that the dyes are desorbed from the hydrogel. Furthermore, the dyes can be sensitively detected by the electrochemical sensor with a low detection limit (5 mg L^−1^, S/N = 3) and wide linear range (from 5 mg L^−1^ to 750 mg L^−1^) (as shown in Fig. 7), indicating that the electrochemical sensor could be used to monitor dye molecules with reasonable assay performance.

In summary, we demonstrate that the PAAM/PA/PDA hydrogel can be used as high efficiency and pH sensitive broad spectrum adsorbent for organic dyes. The high efficiency of the hydrogel is attributed to the abundant functional groups and the three-dimensional porous structure. The PAAM/PA/PDA hydrogel exhibits high adsorption capabilities for a family of dyes with aromatic structure. The adsorption capacities toward four selected dyes are higher than most of reported adsorbents. In particular, the good regeneration highlights the utility of PAAM/PA/PDA hydrogel for environmental remediation.

## Methods

### Materials and reagents

Acrylamide (AAM) and phytic acid (PA, ca. 50% wt/v in water) were obtained from TCI Development Co., Ltd. (Shanghai, China). Dopamine (DA) was ordered from Alfa Aesar Co. (Massachusetts, USA). N,N′-methylenebis-acrylamide (MBA) was purchased from Beijing Aipoo Hua Mei Biotechnology Co., Ltd. (Beijing, China). N,N,N′,N′-tetramethyldiamine (TEMED) and potassium peroxydisulfate (KPS) were received from Beijing Dingguo Biotechnology Ltd. (Beijing, China). Methyl blue (MB, 98%) [C_37_H_27_N_3_Na_2_, MW: 799.81, λ_max_: 598 nm], methylene blue (YMB, 98%) [C_16_H_18_N_3_OS, MW: 333.6 g mol^−1^, λ_max_: 630 nm], methyl violet (MV, 90%) [C_25_H_30_N_3_Cl, MW: 393.96 g mol^−1^, λ_max_: 584 nm] and neutral red (NR, 100%) [C_15_H_17_ClN_4_, MW: 288.77, λ_max_: 532 nm] were purchased from Acros Organics (Belgium, USA). The molecular structures of these dyes were shown in Table S1. All other reagents (analytical grade) used without further purification were purchased from Beijing Chemical Reagents Co. (Beijing, China). All dye aqueous solutions were prepared by dissolving a certain amounts of dyes in deionized water. Milli-Q water (18.2 MΩ cm) was used in all experiments. The pH value of water was adjusted by 1.0 mol L^−1^ HCl and/or 1.0 mol L^−1^ NaOH.

### Characterization and instruments

The Fourier Transform Infrared (FTIR) spectra were recorded with a Bruker Vertex 70 FTIR spectrometer (Bruker, Germany). The pH value was determined by Orion 4-Star Plus pH/ISE Benchtop Multiparameter Meter (Shanghai, China). The zeta potential of PAAM/PA/PDA hydrogel was determined in the pH range from 1 to 10 using Zetasizer Nano ZS (Malvern, UK). The absorbance of dyes at maximum absorption wavelength were recorded using the XS2 microplate reader (BioTek, USA), and the concentrations of the dyes were defined corresponding to calibration curves, respectively. The differential pulse voltammetry (DPV) measurements were performed on a CHI 660D electrochemical workstation (Shanghai, China) with a desired potential range. A conventional three-electrode system was used with an Ag/AgCl (saturated KCl) electrode as the reference electrode, a platinum sheet as the counter electrode and glassy carbon electrode (GCE, 3 mm in diameter) modified with the PAAM/PA/PDA hydrogel film as the working electrode.

### Dye adsorption and desorption studies

The PAAM/PA/PDA conductive hybrid hydrogel was synthesized according to our previously reported strategy (see Supporting Information 1.1 for details)^21^. Dye adsorption experiments on the PAAM/PA/PDA hydrogel were performed by mixing 8 mL dye solutions (50 mg L^−1^) with 5 mg PAAM/PA/PDA hydrogel in a water bath at desired temperatures (285, 298, 308 and 318 K), respectively. In order to monitor the changed amount of dyes in solution, 20 μL supernatants were continuously taken out from the mixture at appropriate time intervals (0, 2, 5, 8, 10, 15, 20, 30, 45, 60, 90, 120, 180, 240, 300, 400, 600 and 720 min), respectively. After diluted with 180 μL water, the maximum absorbance of supernatants were measured for calculating the adsorbed amounts of dyes. After the adsorption process, the hydrogel was moved out from water solution, transferred into 50 mL desorption solution, incubated for 12 hours and washed with 30 mL water (3 times, 30 minutes every time). After dried at 75 °C, the recovered PAAM/PA/PDA hydrogel was ready for next cycle adsorption. The adsorption-desorption procedure was repeated 7 times.

For studying the effect of pH on adsorption capacity, 8 mL dye solutions (50 mg L^−1^) were incubated with 10 mg PAAM/PA/PDA hydrogel under various pH values (3.0, 4.0, 5.0, 5.5 and 6.0 for NR, 3.5, 4.0, 4.5 and 5.0 for MB, 7.5, 8.5, 9.0, 9.5 and 10.0 for MV, 7.5, 8.5, 9.5 and 10.0 for YMB) at 298 K, respectively. For studying the effect of adsorbent dosage on adsorption capacity, 8 mL dye solutions (50 mg L^−1^) were incubated with different PAAM/PA/PDA hydrogel dosages (2.5, 5.0, 7.5 and 10.0 mg) under optimized pH values (pH 5.5 for NR, 4.5 for MB, 10.0 for MV and 10.0 for YMB) at 298 K, respectively. For studying the effect of dye concentration on adsorption capacity, 8 mL dye solutions with various concentrations (5, 10, 25 and 50 mg L^−1^) were incubated with 5.0 mg PAAM/PA/PDA hydrogel at 298 K, respectively. For studying the effect of temperature on adsorption capacity, 8 mL dye solutions with various concentrations (50 mg L^−1^) were incubated with 5.0 mg PAAM/PA/PDA hydrogel at various adsorption temperature (285, 298, 308 and 318 K), respectively. Other experimental conditions/processes were as same as previously described.

### Electrochemical analysis of dye adsorption and desorption on the PAAM/PA/PDA hydrogel

The PAAM/PA/PDA hydrogel modified GCE (termed as PAAM/PA/PDA/GCE) was prepared according to our previously reported strategy (see Supporting Information 1.2 for details)^21^. For dye adsorption, the PAAM/PA/PDA/GCE was immersed into 200 μL dye solutions with various concentrations under suitable pH value (pH 4.5 for MB, pH 10.0 for YMB, pH 10.0 for MV and pH 5.5 for NR) for 300 minutes to form dyes-PAAM/PA/PDA/GCEs. After that, the dyes-PAAM/PA/PDA/GCEs were washed three times by 5 mL water with suitable pH value (pH 4.5 for MB, pH 10.0 for YMB, pH 10.0 for MV and pH 5.5 for NR). Finally, the dyes-PAAM/PA/PDA/GCEs were transferred into 0.1 mol L^−1^ KCl solutions with suitable pH values (pH 4.5 for MB, pH 10.0 for YMB, pH 10.0 for MV and pH 5.5 for NR), and the DPV measurements were immediately recorded. For dye desorption analysis, the dyes-PAAM/PA/PDA/GCEs were immersed into 50 mL water with suitable pH value (pH 11.0 for MB, pH 2.0 for YMB, pH 2.0 for MV and pH 2.0 for NR) at room temperature, and the DPV measurements were obtained.

## Electronic supplementary material

Supporting Information
