# Supplementary material for: Polyacrylamide-Phytic Acid-Polydopamine Conducting Porous Hydrogel for Efficient Removal of Water-Soluble Dyes
Source: Sci Rep. 2017 Aug 11;7:7878. doi: 10.1038/s41598-017-08220-6 (PMC5554154; doi:10.1038/s41598-017-08220-6)
Supplement: Supplementary file 1 — Supporting Information [file 41598_2017_8220_MOESM1_ESM.pdf]

Supporting Information

Polyacrylamide-Phytic Acid-Polydopamine Conducting Porous Hydrogel for Efficient Removal of Water-Soluble Dyes

Zhen Zhao,[1, 2] Leijiao Li,[3] Girma Selale Geleta,[1, 2] Lina Ma,[1] and Zhenxin Wang*,[1]*

[1]State Key Laboratory of Electroanalytical Chemistry, Changchun Institute of Applied Chemistry, Chinese Academy of Sciences, Changchun 130022, P. R. China, [2]University of Chinese Academy of Sciences, Beijing 100049, P. R. China, [3]State Key Laboratory of Rare Earth Resource Utilization, Changchun Institute of Applied Chemistry, Chinese Academy of Sciences, Changchun, 130022, P. R. China.

Correspondence should be addressed to malina@ciac.ac.cn (L. N. Ma) and wangzx@ciac.ac.cn (Z. X. Wang).

Number of pages: 20; Number of tables: 4; Number of figures: 11

1 Experimental section

1.1 Synthesis of the PAAM/PA/PDA hydrogel

The PAAM/PA/PDA conductive hybrid hydrogel was prepared according to the literature.[S1] In brief, 667 μL AAM (6.0 mol L^{-1}), 250 μL PA (pH 7.5, 0.54 mol L^{-1}), 250 μL MBA (0.02 mol L^{-1}, chemical cross-linker) and 250 μL water were mixed with agitating. After de-aired by Ar gas for 10 min, 410 μL DA (0.17 mol L^{-1}) was added to the mixture and ultrasonicated for 5 min. Subsequently, 85.20 mg potassium peroxodisulfate (KPS, initiator) was added to the mixture. After ultrasonicated for 15 min, 0.31 μL TEMED (accelerator) was added, and then the mixture were immediately injected into a mode for further experiments.

1.2 Fabrication PAAM/PA/PDA hydrogel modified electrode

Glassy carbon electrode (GCE) was sequentially polished carefully to a mirror-like surface with 0.3 and 0.05 μm Al_2O_3 powder on a microcloth for 5 min, and then ultrasonicated in water, ethanol and water for 1 min to remove the residual Al_2O_3 powder, respectively. After dried by N_2 air, 5 μL GA (0.5%, w/v) was dropped on the working electrode and dried at 37 °C for 30 min. Subsequently, 5 μL PAAM, PA and PDA mixture was pipetted onto the GA modified electrode and placed at room temperature for 45 min before aggregating into colloid (GA/GCE). This PAAM/PA/PDA hydrogel modified GA/GCE named as PAAM/PA/PDA hydrogel-based electrochemical sensor.

1.3 Swelling experiments

For obtaining the initial weight (W_0), the hydrogels were dried at 75 ºC under vacuum for 2 h. Then, the hydrogels were immersed in H_2O at room temperature. The wet weights (W_t) of hydrogels were recorded at desired times. The swelling ratio (S_W) was defined as: $S_W = (W_t-W_0)/W_0 \times 100\%$. Hydrogels were tested in groups of three.

2 Theoretical background

2.1 Adsorption analysis

On the basis of known dye concentration, we could calculate the average ε from Beer's law.

$$A = \varepsilon bC$$

A is absorbance, ε (L mg^{-1} cm^{-1}) is the molar absorptivity, *b* (cm) is 1, *C* (mg L^{-1}) is concentration of dye samples. Then, from the recorded *A*, known average ε, and *b* we could calculate C_t. The amount of dyes attached onto PAAAM/PA/PDA hydrogel was calculated according to the mass balance equation as

$$q_t = \frac{V(C_0 - C_t)}{W}$$

q_t is the amount of dyes absorbed on PAAM/PA/PDA hydrogel (mg g^{-1}) at the fixed time intervals; C_0 (mg L^{-1}) and C_t (mg L^{-1}) are concentrations of dye in aqueous solution at the initial and t time, respectively; *V* (L) is the employed dyes volume; and *W* (g) is the weight of the used PAAM/PA/PDA hydrogel.

2.2 Kinetic analysis of adsorption

Kinetic analysis of the dye adsorption is performed at 285, 298, 308 and 318 K. Pseudo-first, pseudo-second order model is used. These models are commonly used to describe the adsorption kinetics on solid sorbents. Parameters of the kinetic models were taken from experimental data and linear curve-fitting procedure is used.

2.2.1 Pseudo first-order model

Pseudo-first-order model of Lagerergren is widely used in lower concentration of solute from liquid solution. The pseudo-first-order kinetic equation is written in differentiate form.

$$\frac{dq_t}{dt} = k_1(q_e - q)$$

q_t (mg g^{-1}) is the amount of adsorbed dyes on PAAM/PA/PDA, at any time t (min); q_e (mg g^{-1}) is the amount of adsorbed dyes on PAAM/PA/PDA hydrogel at equilibrium. k_1 is the rate constant of pseudo-first-order adsorption (min^{-1}).

After definite intergradations, the equation becomes

$$\ln(q_e - q_t) = \ln q_e - k_1 t$$

The plot of $\ln(q_e - q_t)$ versus t should give a straight line with slope -k_1 and intercept $\ln q_e$ if the adsorption kinetics follows pseudo first-order model. Table S2 show the correlation coefficient values for adsorption dyes on PAAM/PA/PDA hydrogel varies from 0.5651 to 0.7649, from 0.8008 to 0.8206, from0.7305 to 0.8616, and from 0.7611 to 0.8297, for NR, MB, MV and YMB, respectively. These results show that experimental data does not agree with the pseudo first-order kinetic model.

2.2.2 Pseudo second-order model

The pseudo second-order model equation on adsorption equilibrium can be expressed as following

$$\frac{dq_t}{dt} = k_2(q_e - q)^2$$

k_2 is the rate constant of pseudo second-order adsorption. Integrated form becomes

$$\frac{t}{q} = \frac{1}{k_2 q_e^2} + \frac{t}{q_e}$$

The plot of t/q versus t should give a straight line with slop $1/q_e$ and intercept $1/k_2 q_e^2$ if the adsorption kinetics follows pseudo second-order model.

2.2.3 Adsorption mechanism-Intra-particle diffusion model

Weber-Morris plot shows adsorption capacity (q_t) versus $t^{0.5}$ relation with linearity. It can be expressed by

$$q_t = k_i\sqrt{t} + C$$

where k_i is an intra-particle diffusion parameter (mg g^{-1} min $^{-0.5}$) and C is the intercept. If the intercept is 0, intra-particle diffusion is the only process mechanism dominant in adsorption. If not, it will have complex mechanism with others.

2.3 Thermodynamic analysis

From kinetic analysis, the pseudo second-order is the dominant adsorption kinetics. We estimate the activation energy and Arrhenius factor of dyes adsorption on PAAM/PA/PDA hydrogel with Arrhenius equation using k_2.

$$lnk_2 = lnk_0 - \frac{E_a}{RT}$$

E_a is activation energy (J mol^{-1}); k_2 is the rate constant of adsorption (g mg^{-1} min^{-1}); R is gas constant (8.314 J K^{-1} mol^{-1}); T is the solution temperature (K). By plotting the experiment data, lnk_2 versus $1/T$, we got slope $-\frac{E_a}{R}$ and intercept lnk_0.

Enthalpy of activation ($\Delta H^{\#}$) can be derived by Eyring equation. Originally Eyring equation is derived from first-order kinetic process, but it can be used for second-order kinetic process if it is spatial symmetry.

$$ln\frac{k_2}{T} = ln\frac{k_b}{h} + \frac{\Delta S^{\#}}{R} - \frac{\Delta H^{\#}}{RT}$$

Where k_b is Boltzmann's constant (1.381 × 10^{-23} J K^{-1}); h is Plank's constant (6.626×10^{-34} J s); k_2 is the rate constant of adsorption (g mg^{-1} min^{-1}); R is the gas constant (J K^{-1} mol^{-1}); T is the solution temperature (K). Figure 3b shows a plot of $\ln\frac{k_2}{T}$ versus $\frac{1}{T}$ with slope $-\frac{\Delta H^{\#}}{R}$.

3 Tables

Table S1. The structure of dye molecule and PAAM/PA/PDA hydrogel

Material	Structure
PAAM/PA/PDA hydrogel	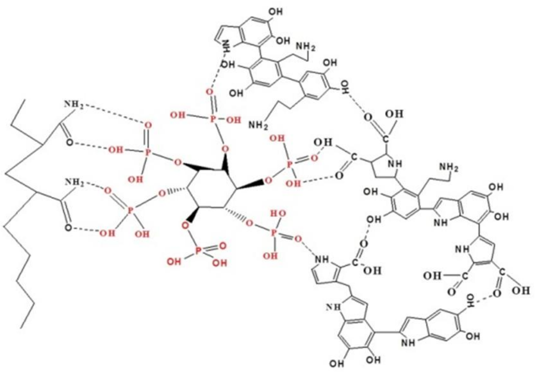
MB	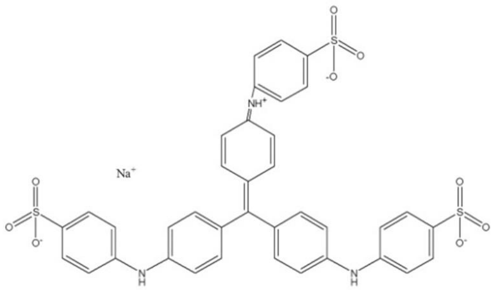
NR	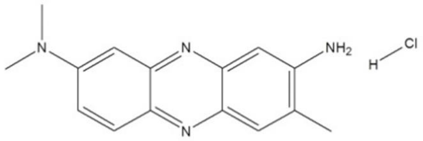
MV	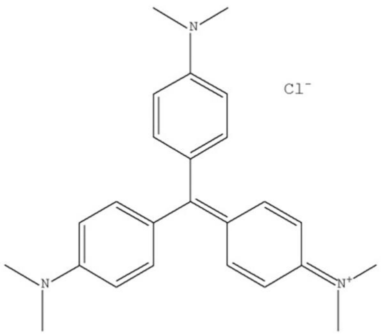
YMB	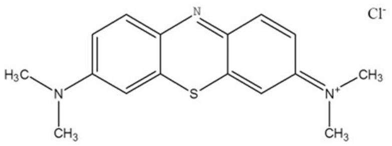

Table S2. Coefficients of a Pseudo Second-Order Diffusion Model for MB and MV Adsorption on the PAAM/PA/PDA hydrogel

Dyes	T (K)	$Q_{e,exp}$ (mg g^{-1})	$Q_{e,cal}$ (mg g^{-1})	k_2 (g mg^{-1} min^{-1})	R^2
NR	285	550.59	540.54	0.00185	0.9979
	298	566.61	568.18	0.00176	0.9996
	308	575.76	571.43	0.00175	0.9999
	318	584.58	581.40	0.00172	0.9999
MB	285	332.06	333.30	0.00300	0.9999
	298	381.35	380.23	0.00263	0.9997
	308	391.51	392.17	0.00255	0.9999
	318	405.99	406.50	0.00246	0.9998
MV	285	485.92	476.19	0.00210	0.9958
	298	529.96	520.83	0.00192	0.9958
	308	568.68	568.18	0.00176	0.9991
	318	625.97	628.93	0.00159	0.9996
YMB	285	280.97	277.00	0.00361	0.9979
	298	327.56	319.49	0.00313	0.9911
	308	341.82	335.57	0.00298	0.9910
	318	350.67	344.83	0.00290	0.9913

Table S3. Coefficients of a pseudo first-order diffusion model for MB and MV adsorption on the PAAM/PA/PDA hydrogel

Dyes	T (K)	$Q_{e,exp}$ (mg g^{-1})	$Q_{e,cal}$ (mg g^{-1})	k_1 (min^{-1})	R^2
NR	285	550.59	163.65	0.01073	0.7649
	298	566.61	155.65	0.00429	0.6436
	308	575.76	84.12	0.00558	0.5651
	318	584.58	58.51	0.00968	0.6987
MB	285	332.06	67.34	0.01182	0.8206
	298	381.35	76.50	0.01085	0.8024
	308	391.51	60.85	0.01189	0.8008
	318	405.99	117.22	0.01933	0.8878
MV	285	485.92	257.60	0.00728	0.7305
	298	529.96	302.77	0.00813	0.7353
	308	568.68	281.30	0.00901	0.7670
	318	625.97	285.72	0.00965	0.8616
YMB	285	280.97	137.02	0.01446	0.7611
	298	327.56	194.98	0.01103	0.8185
	308	341.82	224.98	0.01251	0.7917
	318	350.67	229.95	0.01192	0.8297

Table S4. Coefficients of an Intraparticle Diffusion Model forMB and MV Adsorption on the PAAM/PA/PDA hydrogel

Dyes	T (K)	k_{i1} (g mg^{-1} $min^{-0.5}$)	k_{i2} (g mg^{-1} $min^{-0.5}$)	R_1^2	R_2^2
NR	285	3.7067	7.3205	0.9544	0.9969
	298	15.1659	4.5735	0.9699	0.9466
	308	12.4704	1.4883	0.9546	0.9255
	318	3.5921	1.6204	0.8801	0.9764
MB	285	36.3053	2.0759	0.8817	0.8102
	298	37.5330	2.1147	0.8239	0.9215
	308	40.9202	1.4653	0.7633	0.9447
	318	41.1573	2.5589	0.8706	0.9457
MV	285	5.3532	8.0412	0.9269	0.9721
	298	8.8193	8.7774	0.8577	0.9798
	308	14.3820	6.1228	0.9745	0.9033
	318	21.2511	5.1760	0.9755	0.8806
YMB	285	8.2392	3.27010	0.9491	0.9807
	298	8.6259	7.76560	0.9961	0.9868
	308	9.1223	8.13211	0.9929	0.9940
	318	11.0109	8.07457	0.9679	0.9915

4 Figures

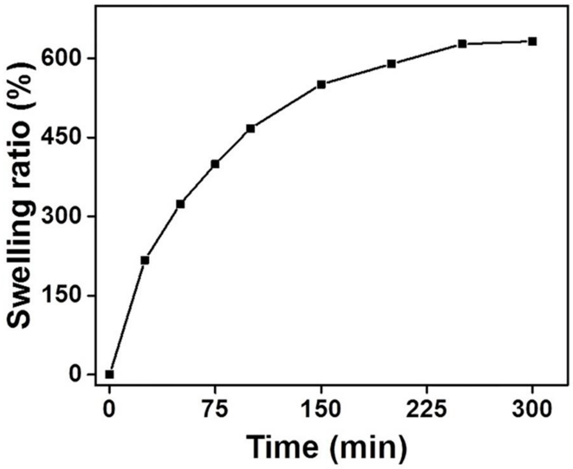

Figure S1 The swelling properties in H_2O of PAAM/PA/PDA hydrogel.

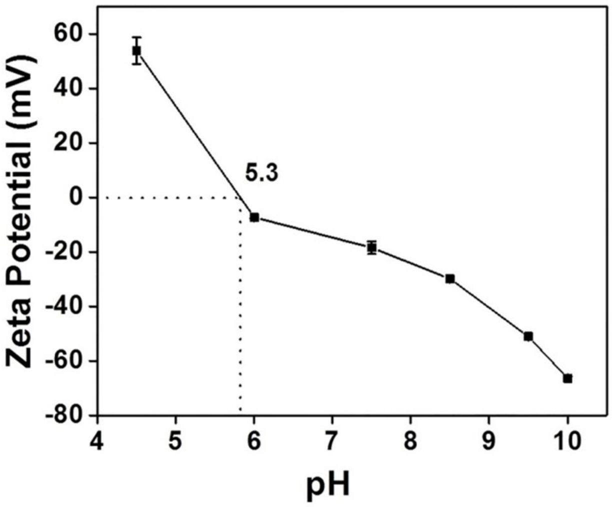

Figure S2 Effects of pH values on zeta potential of PAAM/PA/PDA hydrogel.

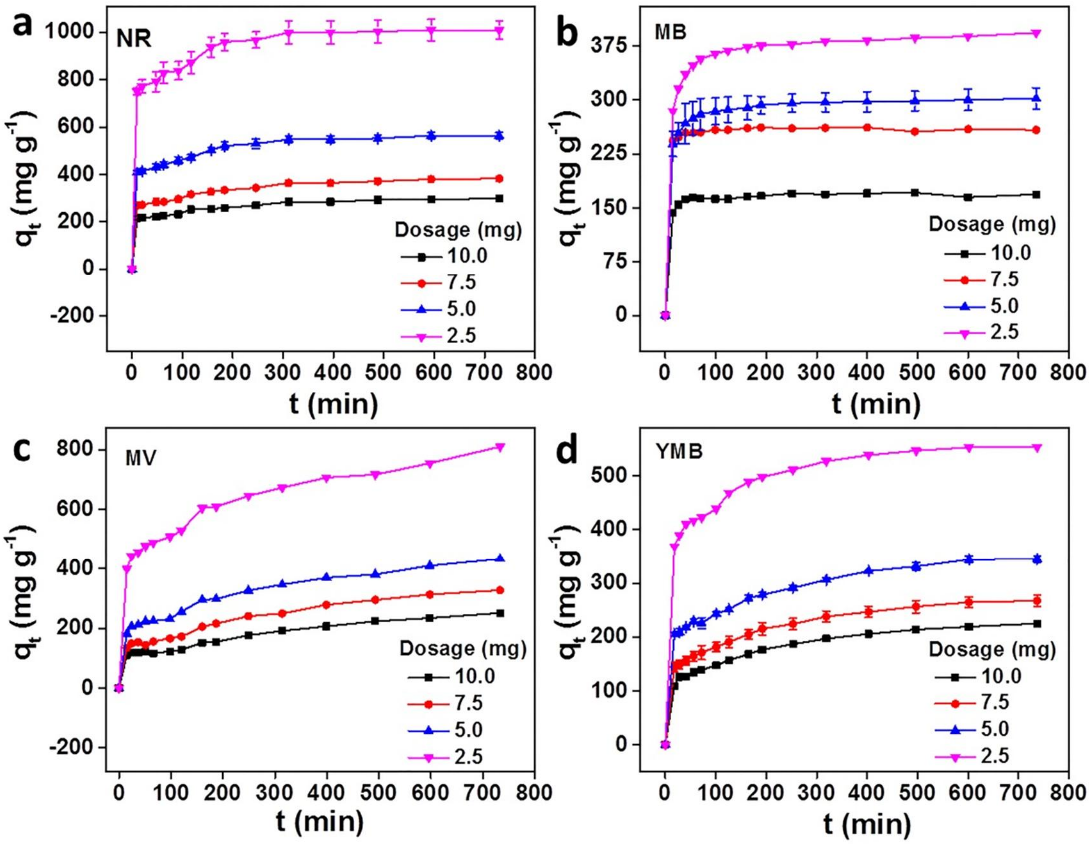

Figure S3 The effect of adsorbent dosage on the adsorption of PAAM/PA/PDA hydrogel to (a) NR, (b) MB, (c) MV and (d) YMB, respectively.

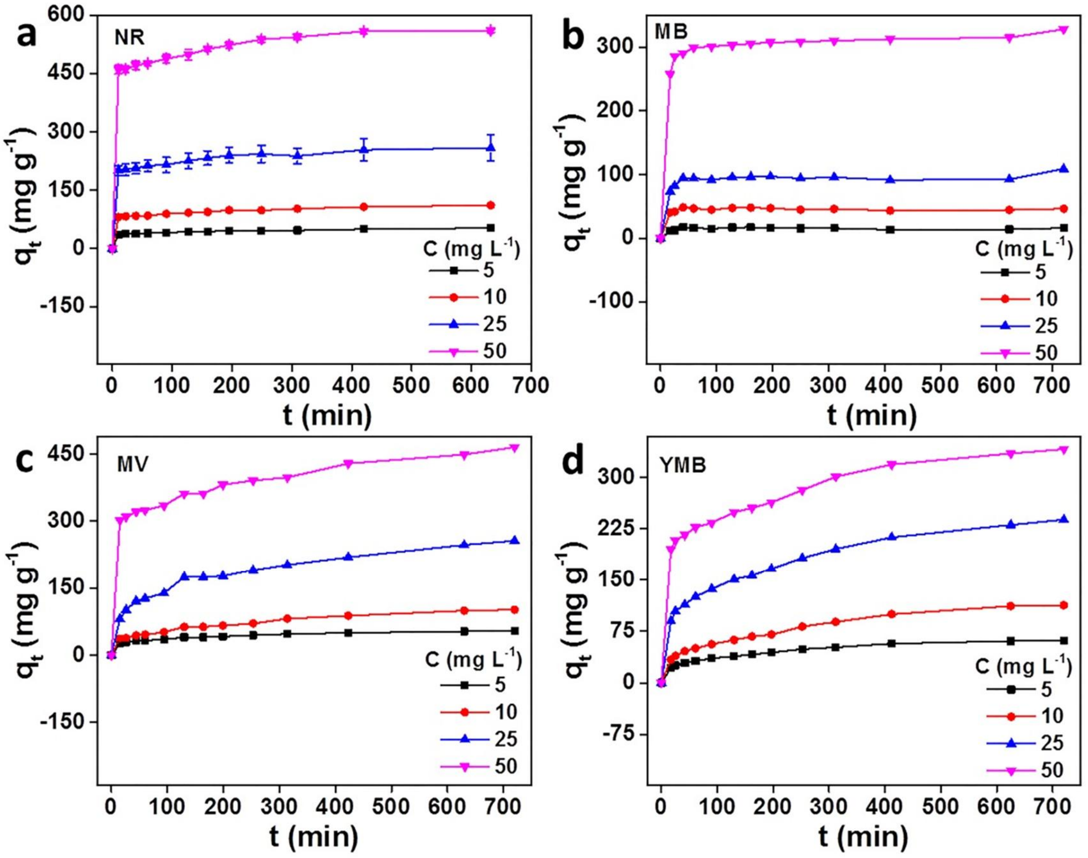

Figure S4 The effect of initial dye concentration on the adsorption of PAAM/PA/PDA hydrogel to (a) NR, (b) MB, (c) MV and (d) YMB, respectively.

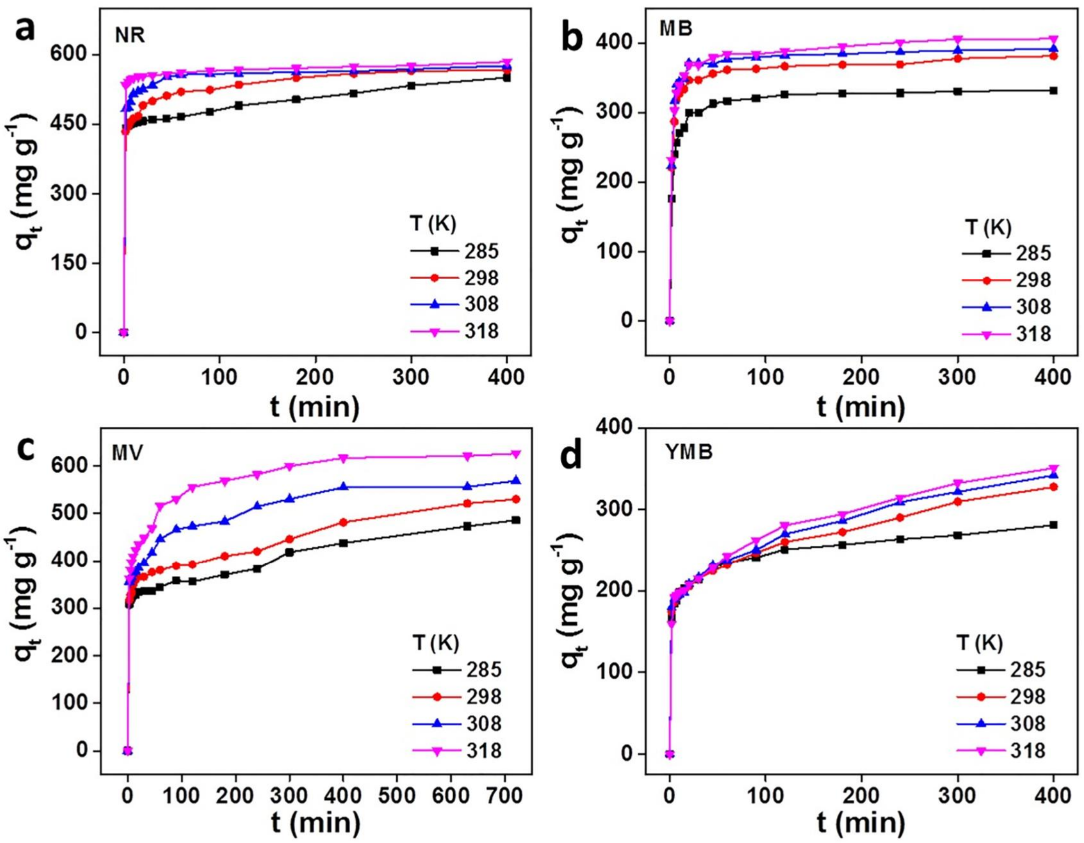

Figure S5 The effect of temperature on the adsorption of PAAM/PA/PDA hydrogel to (a) NR, (b) MB, (c) MV and (d) YMB, respectively.

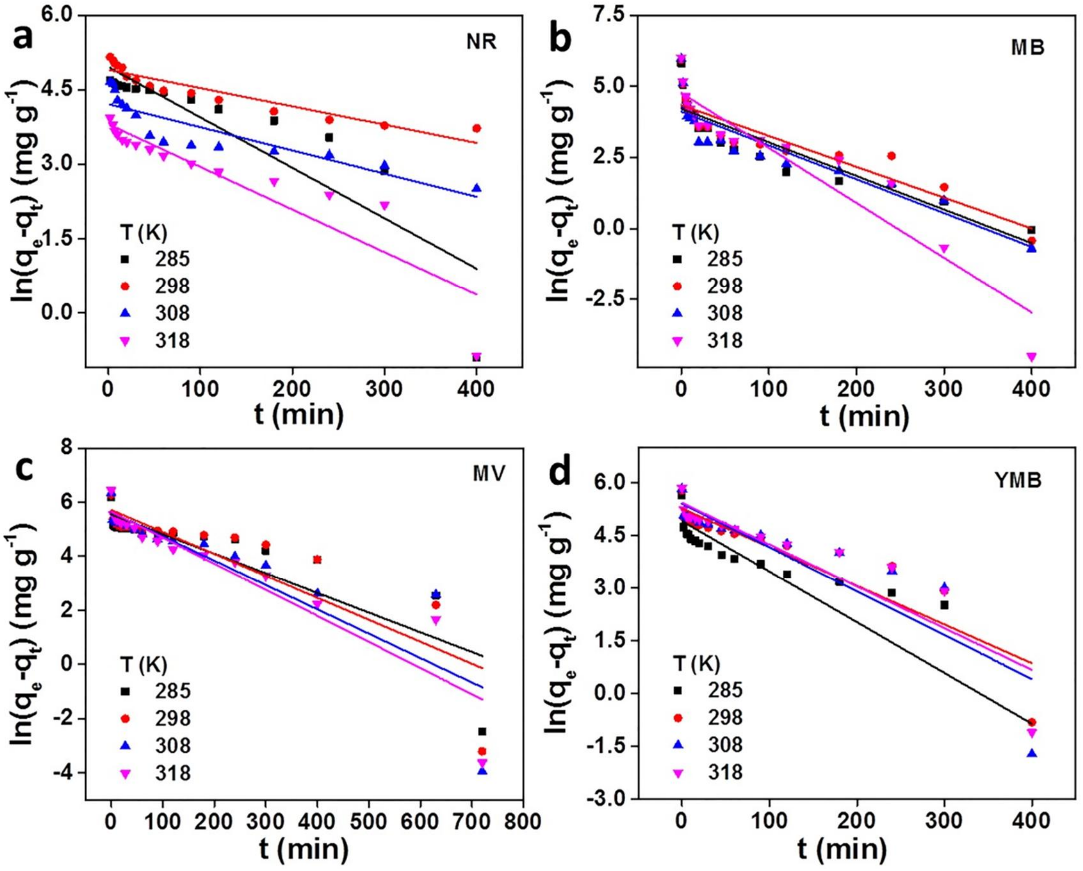

Figure S6 Plots of pseudo first-order models for (a) NR, (b) MB, (c) MV and (d) YMB on PAAM/PA/PDA hydrogel.

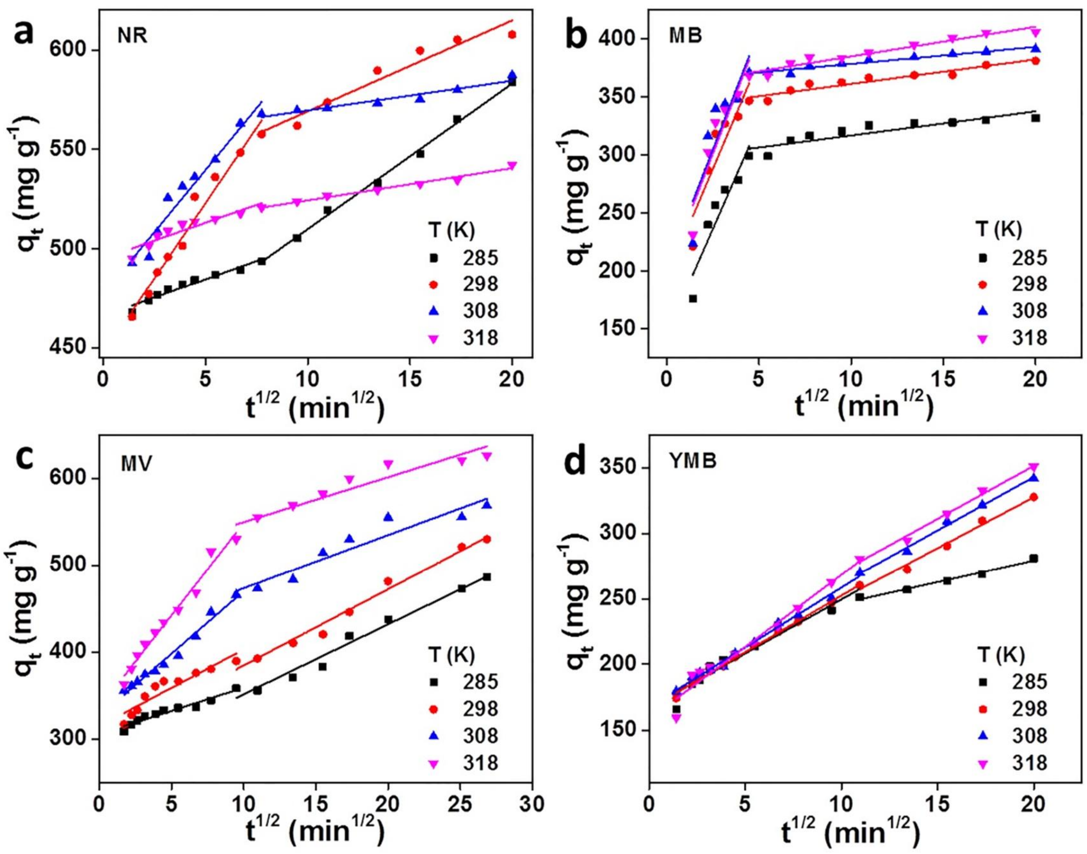

Figure S7 Plots of intra-particle diffusion models for (a) NR, (b) MB, (c) MV and (d) YMB on PAAM/PA/PDA hydrogel.

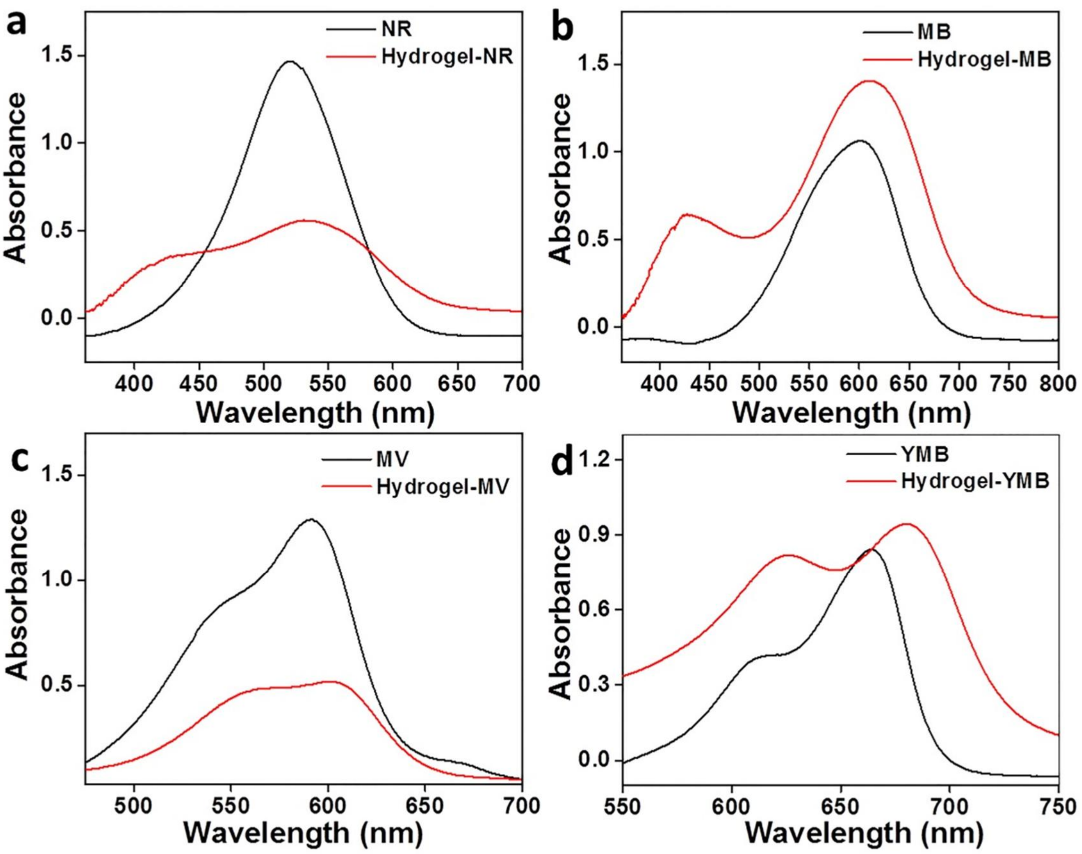

Figure S8 UV-visible absorption spectra of (a) NR and NR-PAAM/PA/PDA hydrogel, (b) MB and MB-PAAM/PA/PDA hydrogel, (c) MV and MV-PAAM/PA/PDA hydrogel, and (d) YMB and YMB-PAAM/PA/PDA hydrogel.

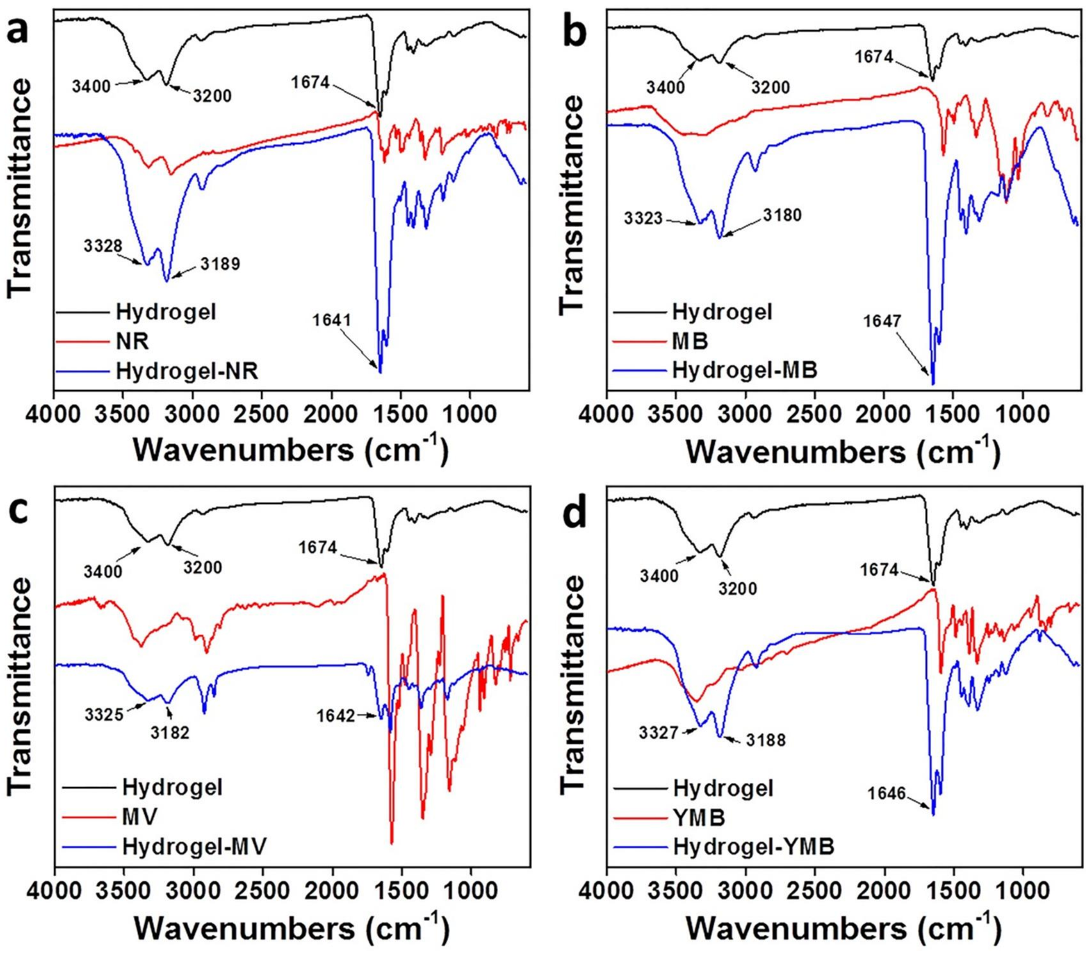

Figure S9 FTIR spectra of (a) NR and NR-PAAM/PA/PDA hydrogel, (b) MB and MB-PAAM/PA/PDA hydrogel, (c) MV and MV-PAAM/PA/PDA hydrogel, and (d) YMB and YMB-PAAM/PA/PDA hydrogel.

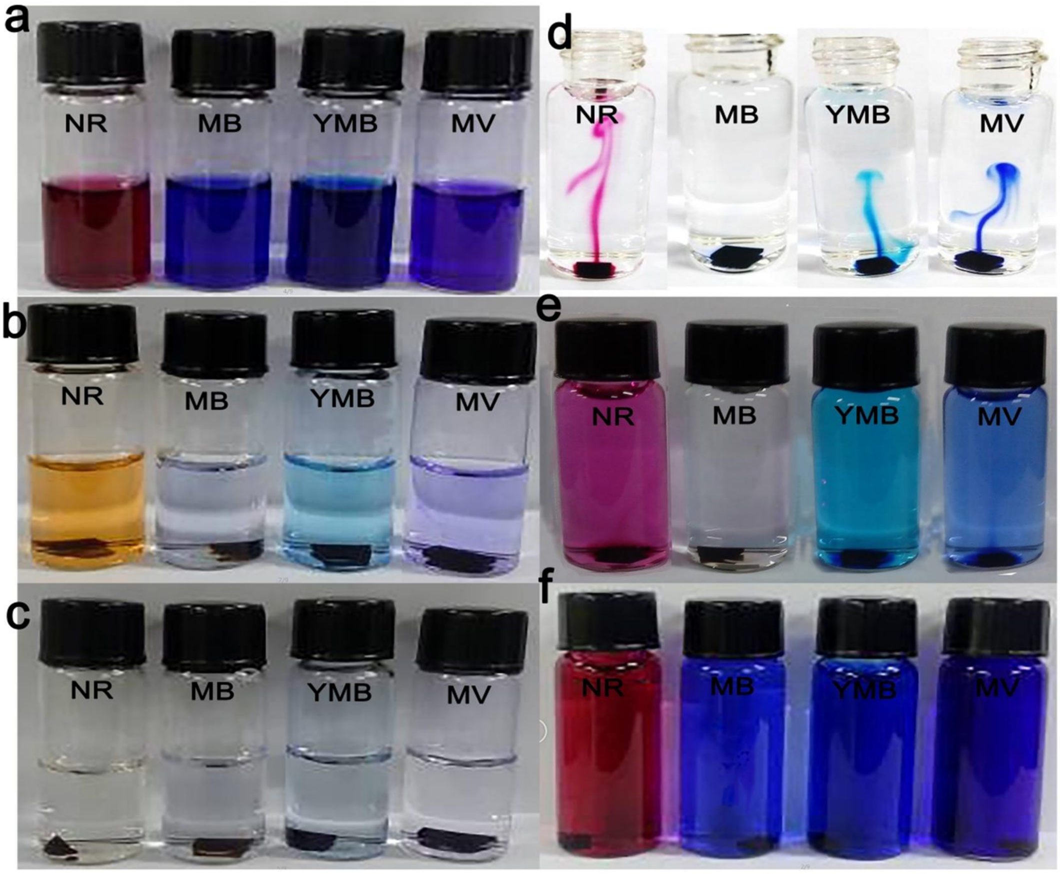

Figure S10 Digital images of dye solutions correspond to different adsorption-desorption process. adsorption process (a) the original dye solution, after adsorbed by PAM/PA/PDA hydrogel for (b) 12 hours and (c) 24 hours; and desorption process desorbed (d) 5 min, (e) 30 min, (f) 24 hours after adjusting the pH to 5.5, 4.5, 10.0 and 10.0 corresponding to NR, MB, MV and YMB, respectively.

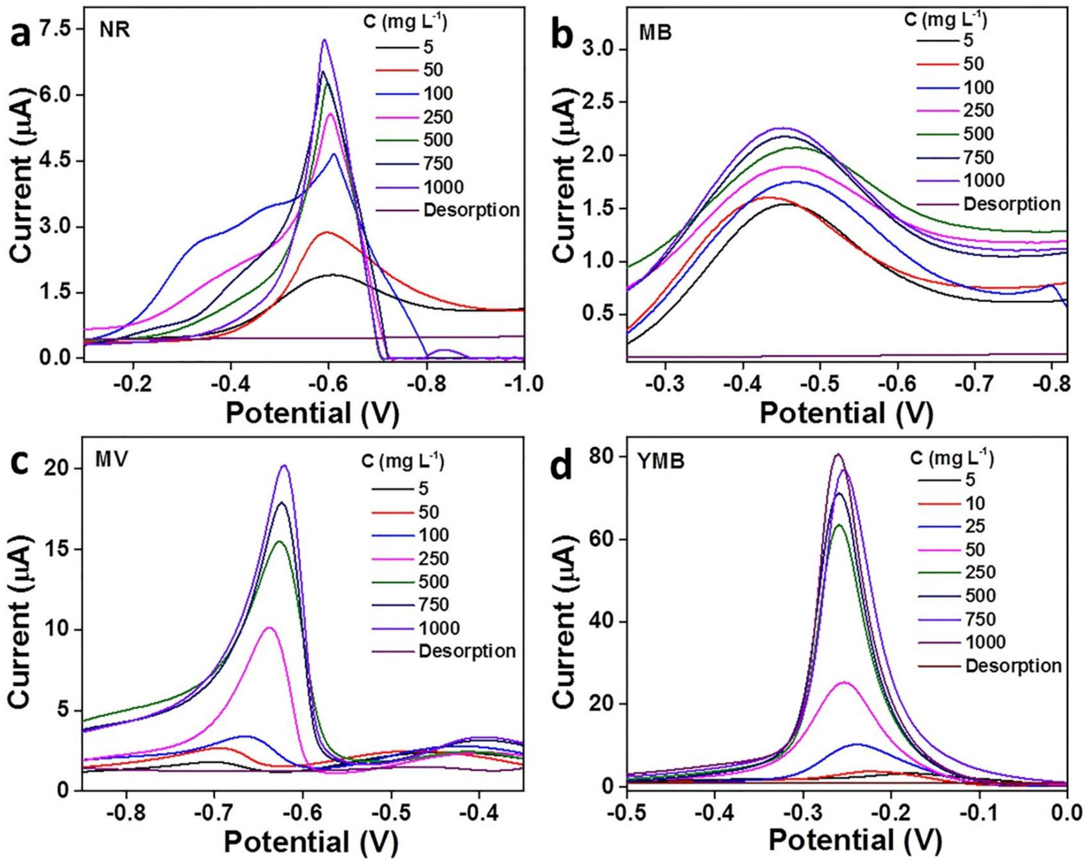

Figure S11 DPV curves of the PAAM/PA/PDA hydrogel-based electrochemical sensors correspond to various concentrations of (a) NR at pH 5.5, (b) MB at pH 4.5, (c) MV at pH 10.0 and (d) YMB at pH 10.0, respectively.

5 Reference

S1. Zhao Z, Chen H, Zhang H, Ma L, Wang Z. *Biosens. Bioelectron.*, 2017, 91, 306-312.
